# Editorial: Captive animal behavior: Individual differences in learning and cognition, and implications on animal welfare

**DOI:** 10.3389/fvets.2022.1102122

**Published:** 2022-12-02

**Authors:** Jan Langbein, Christian Nawroth

**Affiliations:** Institute of Behavioural Physiology, Research Institute for Farm Animal Biology, Dummerstorf, Germany

**Keywords:** companion animals, farm animals, livestock, learning and cognition, personality

To provide adequate welfare for animals in captivity, it is important to consider not only the needs of the species, but also those of the individual. In this context, knowledge about individual differences in learning and cognitive functioning are of particular importance, as it can help to assess the extent to which captive animals are able to adapt and respond to changing housing conditions.

In the last decades, individual differences in learning and cognition have been studied systematically across a wide range of taxa (1). However, the underlying factors that cause this variation, as well as its potential welfare consequences, are still under debate (2). While ultimate factors tend to play a minor role in explaining behavioral variation in captive animals, a variety of proximate factors could be responsible for the individual variation we see in animals' performance in learning and cognitive tasks (Finkemeier et al.). These factors include a variety of genetic and environmental components, ranging from breed or feeding type, to housing conditions (single vs. group housed), to idiosyncrasies of different research sites (3–5). The observation of robust intra-species variation in behavior under identical environmental conditions has led to a significant increase in research on inter-individual behavioral variation, often coined as personality, in many animal taxa, especially in the field of behavioral ecology (6). This behavioral variation can be observed in levels of activity, as well as exploratory and social behaviors, beside others. While the influence of genetic, physiological and behavioral factors on individual response patterns in farm, laboratory, and zoo animals has received considerable attention in recent years, few studies have addressed the role of these traits in predicting inter-individual differences in learning and cognition.

The objective of this Research Topic was to promote interdisciplinary research approaches on the link between individual variation in genetics, physiology and behavior, and learning and cognition—ranging from fields such as developmental psychology to applied ethology and addressing this variation in animals under human care, with particular emphasis on farm, companion and zoo animals. The manuscripts included in this Research Topic have examined the impact of genetics, neurotransmitters, hormones, critical life stages, and certain personality traits on learning and cognitive phenomena such as cooperation and self-control and range from farm animals (goat, pig, and chicken) to companion (dog, horse) and laboratory animals (rat) as studied species.

Studies in our Research Topic often focused on the association between different behavioral parameters and inter-individual differences in learning and other cognitive phenomena. In a study in goats, Finkemeier et al. investigated the relationship between distinctive personality traits and discrimination learning. Stability in the personality trait boldness was found to have an impact on learning performance in a visual reversal-learning task, with less bold goats performing better than bolder ones. These results support the general hypothesis that proactive animals tend to stick to once-learned routines and to react less flexibly to changing stimulus combinations (7). To study cooperative behavior in pigs, Rault et al. developed an ecologically relevant feeding paradigm, the so-called “joint log-lift task”. To complete the task, two pigs must cooperate in lifting a log to receive a reward. While kinship had no influence on the cooperation behavior of individual dyads, inter-individual differences in sociability influenced the willingness to cooperate in pigs. The relationship between social competence and the impact of intranasal oxytocin on social behavior of dogs was studied by Turcsán et al. While oxytocin has been reported to have a general positive effect on social behavior, intranasal administration of oxytocin in this study increased social behavior in dogs toward humans only in animals that showed already a low baseline performance of interacting with humans. This indicates that the effect of oxytocin on social behavior is dependent on personality traits and the specific context. A study by Brucks et al. investigated self-control in horses. They found that horses wait until a maximum delay of 60 s to receive a highly valued reward rather than to get an immediately available reward of lower quality. While horses fed hay *ad libitum* instead of receiving a restricted diet achieved higher delay times, the trainability or patience of the horses had no influence on the maximum delay level.

Two of the submitted studies aimed to establish a relationship between different genetic predispositions and learning performance or flexibility in learning behavior. To test whether animals bred for high productivity have lower learning performance, Nawroth et al. compared the performance of dwarf goats and dairy goats in a visual discrimination and reversal-learning task. The results suggest that selection for high performance may have negatively affected the goats' behavioral flexibility with dwarf goats outperforming dairy goats in reversal learning. Dudde et al. investigated the role of the serotonin transporter (5-HTT) on anxiety and learning performance in chicken. Chicken from selection lines with different 5-HTT polymorphisms were tested with regard to their fearfulness and performance in a simple discrimination task. Chicken with reduced 5-HTT expression showed increased anxiety-like behavior, as has also been demonstrated in humans. However, and in contrast to human research, animals with reduced 5-HTT expression were also the slowest learners compared to hens with moderate or high expression.

Finally, three of the included studies addressed the effects of ontogeny or specific critical life stages on the cognitive abilities in chicken and pig. Garnham et al. investigated the relationship between affective states and inhibitory control in the red jungle fowl. Inhibitory control was measured using a detour task, while measures for affective states derived from the tonic immobility test and a cognitive judgement bias test. While inhibitory control was associated with affective states in younger chicks, no such association was found in older hens. The study shows that the link between affective states and inhibitory control can change during ontogeny. In another study in pigs by Bushby et al., a spatial judgement bias task was used to investigate the extent to which gestation affects the mood of pregnant sows. The reaction of gilts to ambiguous probe locations were tested at different stages of gestation. The results suggest that the mood of pigs can change during pregnancy, which could have an impact on the assessment of the welfare of captive multiparous animals. A study by Nagano examined that modified training to handle a rake-shaped tool in relation to an unreachable reward did not improve the rats' tool manipulation ability.

The studies, summarized in this Research Topic will improve our understanding of the internal and external factors that influence the expression of cognitive abilities in companion, laboratory, and farm animals, and how this in turn can have implications for their welfare.

## Author contributions

Both authors listed have made a substantial, direct, and intellectual contribution to the work and approved it for publication.

## Funding

This work was supported by grants from the Deutsche Forschungsgemeinschaft (DFG, LA 1187/6-1) to JL.

## Conflict of interest

The authors declare that the research was conducted in the absence of any commercial or financial relationships that could be construed as a potential conflict of interest.

## Publisher's note

All claims expressed in this article are solely those of the authors and do not necessarily represent those of their affiliated organizations, or those of the publisher, the editors and the reviewers. Any product that may be evaluated in this article, or claim that may be made by its manufacturer, is not guaranteed or endorsed by the publisher.
